# 4-Bromo­methyl-7-methyl-6,8-dinitro­coumarin

**DOI:** 10.1107/S160053680903596X

**Published:** 2009-09-12

**Authors:** Ramakrishna Gowda, Ganesh N. Alawandi, Manohar V. Kulkarni, K. V. Arjuna Gowda

**Affiliations:** a Department of Physics, Goverment College for Women, Kolar 563 101, Karnataka, India; b Department of Chemistry, Karnatak University, Dharwad 580 003, Karnataka, India; c Department of Physics, Goverment First Grade College, K.R. Pura, Bangalore 560 036, Karnataka, India

## Abstract

The crystal structure of the title compound, C_11_H_7_BrN_2_O_6_, establishes the substitution positions of the nitro groups from the nitration reaction of 7-methyl-4-bromo­methyl coumarin. The mean planes of the nitro groups form dihedral angles of 43.9 (8) and 52.7 (10)° with the essentially planar [maximum deviation 0.031 (6) Å] benzopyran ring system.

## Related literature

For background information on the nitration of coumarin compounds, see: Kulkarni *et al.* (1983 ▶); Clayton *et al.* (1910 ▶). For a related structure, see: Vasudevan *et al.* (1990 ▶). For *ab initio* calculations on 6-methyl-4-bromo­methyl­coumarins, see: Sortur *et al.* (2006 ▶).
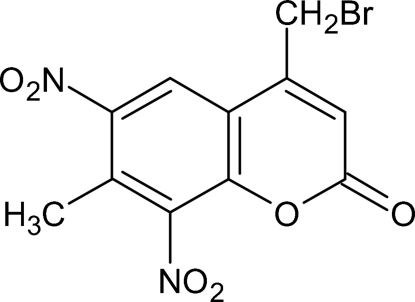


## Experimental

### 

#### Crystal data

C_11_H_7_BrN_2_O_6_
*M* *_r_* = 343.09
Orthorhombic, 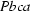

*a* = 8.122 (2) Å
*b* = 11.091 (4) Å
*c* = 27.723 (6) Å
*V* = 2497.3 (12) Å^3^
*Z* = 8
Mo *K*α radiation
μ = 3.32 mm^−1^
*T* = 294 K
0.2 × 0.2 × 0.1 mm

#### Data collection

Enraf–Nonius CAD-4 diffractometer
Absorption correction: ψ scan (North *et al.*, 1968 ▶) *T*_min_ = 0.520, *T*_max_ = 0.72
2196 measured reflections
2196 independent reflections
1148 reflections with *I* > 2σ(*I*)
2 standard reflections frequency: 60 min intensity decay: none

#### Refinement

*R*[*F* ^2^ > 2σ(*F* ^2^)] = 0.060
*wR*(*F* ^2^) = 0.171
*S* = 1.05
2196 reflections
182 parameters
H-atom parameters constrained
Δρ_max_ = 0.63 e Å^−3^
Δρ_min_ = −0.82 e Å^−3^

### 

Data collection: *CAD-4 Software* (Enraf–Nonius, 1989 ▶); cell refinement: *CAD-4 Software*; data reduction: *XCAD4* (Harms & Wocadlo, 1995 ▶); program(s) used to solve structure: *SIR92* (Altomare *et al.*, 1994 ▶); program(s) used to refine structure: *SHELXL97* (Sheldrick, 2008 ▶); molecular graphics: *ORTEP-3 for Windows* (Farrugia, 1997 ▶); software used to prepare material for publication: *SHELXL97*.

## Supplementary Material

Crystal structure: contains datablocks global, I. DOI: 10.1107/S160053680903596X/lh2864sup1.cif

Structure factors: contains datablocks I. DOI: 10.1107/S160053680903596X/lh2864Isup2.hkl

Additional supplementary materials:  crystallographic information; 3D view; checkCIF report

## Figures and Tables

**Fig. 1.**
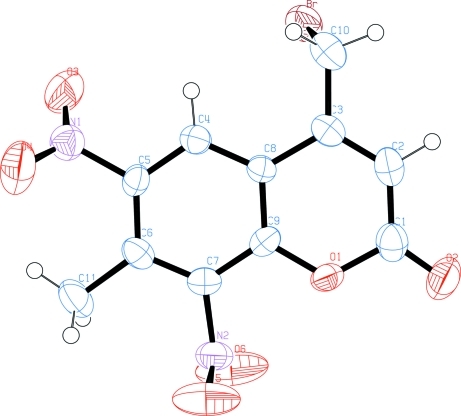
The molecular structure of the title compound, showing 50% probability displacement ellipsoids and the atomic numbering.

## supplementary crystallographic information

### Comment

The molecular structure of the title compound is shown in Fig. 1. The bromomethyl group is twisted is out of the plane of the benzopyran ring as described by the torsion angle of 104.6 (7)° for C2-C3-C10-Br. This is in agreement with the ab initio calculations on 6-methyl-4- bromomethylcoumarins (Sortur *et al.*,  2006). Positions C-6 and C-8 (refers to positions from systematic naming scheme) become activated due to the electron donating methyl group at C-7 and hence nitration occurs at C-6 and C-8 consistent with the title compound which is also in agreement with earlier reports (Clayton, 1910; Kulkarni *et al.*, 1983).

### Experimental

5.06 g of 7-methyl-4-bromomethyl coumarin (0.02 mol) was dissolved in conc. sulfuric acid (10 ml) and treated with a nitrating mixture 15 ml (10 ml H_2_SO_4_ + 5 ml HNO_3_) at ice bath temperatures (273-278K). The reaction mixture was then allowed to stand at room temperature for two hours and the reaction mixture was poured over crushed ice. The separated solid was washed with excess of water, dried and recrystallized from glacial acetic acid. Crystals suitable for diffraction studies were grown by slow evaporation of an ethanol solution of the title compound.

### Refinement

Hydrogen atoms were positioned geometrically with C—H = 0.93-0.97 A° and included in the refinment in a riding-model approximation with *U*_iso_(H) = 1.2 *U*_eq_(C) or 1.5 *U*_eq_(C) for methyl C atoms.

### Crystal data

**Table:**

C_11_H_7_BrN_2_O_6_	*F*(000) = 1360
*M**_r_* = 343.09	*D*_x_ = 1.825 Mg m^−^^3^
Orthorhombic, *P**b**c**a*	Mo *K*α radiation, λ = 0.71069 Å
Hall symbol: -P 2ac 2ab	Cell parameters from 25 reflections
*a* = 8.122 (2) Å	θ = 10–15°
*b* = 11.091 (4) Å	µ = 3.32 mm^−^^1^
*c* = 27.723 (6) Å	*T* = 294 K
*V* = 2497.3 (12) Å^3^	Plate, colourless
*Z* = 8	0.2 × 0.2 × 0.1 mm

### Data collection

**Table:**

Enraf–Nonius CAD-4 diffractometer	1148 reflections with *I* > 2σ(*I*)
Radiation source: fine-focus sealed tube	*R*_int_ = 0.0000
graphite	θ_max_ = 25.0°, θ_min_ = 2.9°
ω–2θ scans	*h* = 0→9
Absorption correction: ψ scan (North *et al.*, 1968)	*k* = 0→13
*T*_min_ = 0.520, *T*_max_ = 0.72	*l* = 0→32
2196 measured reflections	2 standard reflections every 60 min
2196 independent reflections	intensity decay: none

### Refinement

**Table:**

Refinement on *F*^2^	0 restraints
Least-squares matrix: full	H-atom parameters constrained
*R*[*F*^2^ > 2σ(*F*^2^)] = 0.060	*w* = 1/[σ^2^(*F*_o_^2^) + (0.0687*P*)^2^ + 11.8667*P*] where *P* = (*F*_o_^2^ + 2*F*_c_^2^)/3
*wR*(*F*^2^) = 0.171	(Δ/σ)_max_ = 0.001
*S* = 1.05	Δρ_max_ = 0.63 e Å^−^^3^
2196 reflections	Δρ_min_ = −0.82 e Å^−^^3^
182 parameters	

### Special details

**Table:**

Geometry. All e.s.d.'s (except the e.s.d. in the dihedral angle between two l.s. planes) are estimated using the full covariance matrix. The cell e.s.d.'s are taken into account individually in the estimation of e.s.d.'s in distances, angles and torsion angles; correlations between e.s.d.'s in cell parameters are only used when they are defined by crystal symmetry. An approximate (isotropic) treatment of cell e.s.d.'s is used for estimating e.s.d.'s involving l.s. planes.
Refinement. Refinement of *F*^2^ against ALL reflections. The weighted *R*-factor *wR* and goodness of fit *S* are based on *F*^2^, conventional *R*-factors *R* are based on *F*, with *F* set to zero for negative *F*^2^. The threshold expression of *F*^2^ > σ(*F*^2^) is used only for calculating *R*-factors(gt) *etc*. and is not relevant to the choice of reflections for refinement. *R*-factors based on *F*^2^ are statistically about twice as large as those based on *F*, and *R*- factors based on ALL data will be even larger.

### Fractional atomic coordinates and isotropic or equivalent isotropic displacement parameters (Å^2^)

**Table:**

	*x*	*y*	*z*	*U*_iso_*/*U*_eq_	
C1	0.7703 (11)	0.2886 (7)	0.6819 (3)	0.047 (2)	
C2	0.9438 (10)	0.2873 (7)	0.6922 (3)	0.045 (2)	
H3	0.9856	0.3447	0.7134	0.054*	
C3	1.0484 (9)	0.2073 (6)	0.6727 (3)	0.0380 (18)	
C4	1.0779 (9)	0.0341 (6)	0.6146 (3)	0.0364 (18)	
H5	1.1898	0.0281	0.6213	0.044*	
C5	1.0085 (9)	−0.0431 (6)	0.5812 (2)	0.0348 (17)	
C6	0.8400 (9)	−0.0445 (6)	0.5709 (3)	0.0395 (19)	
C7	0.7498 (9)	0.0406 (6)	0.5954 (2)	0.0358 (16)	
C8	0.9860 (8)	0.1194 (6)	0.6381 (3)	0.0326 (17)	
C9	0.8194 (9)	0.1222 (6)	0.6280 (3)	0.0339 (17)	
C10	1.2259 (10)	0.2058 (7)	0.6867 (3)	0.049 (2)	
H11A	1.2542	0.2804	0.7030	0.059*	
H11B	1.2942	0.1987	0.6581	0.059*	
C11	0.7574 (12)	−0.1318 (7)	0.5373 (3)	0.057 (2)	
H12A	0.8385	−0.1842	0.5234	0.085*	
H12B	0.7020	−0.0882	0.5122	0.085*	
H12C	0.6787	−0.1790	0.5550	0.085*	
N1	1.1229 (9)	−0.1278 (6)	0.5564 (3)	0.0483 (17)	
N2	0.5699 (8)	0.0459 (6)	0.5897 (3)	0.0477 (17)	
O1	0.7146 (6)	0.2044 (4)	0.64815 (18)	0.0406 (13)	
O2	0.6689 (8)	0.3553 (6)	0.6980 (2)	0.0649 (18)	
O3	1.2252 (9)	−0.1757 (6)	0.5791 (3)	0.085 (2)	
O4	1.1032 (9)	−0.1384 (6)	0.5134 (3)	0.079 (2)	
O5	0.5137 (9)	0.0940 (7)	0.5564 (3)	0.099 (3)	
O6	0.4882 (8)	0.0020 (9)	0.6212 (3)	0.113 (3)	
Br	1.26518 (11)	0.06847 (7)	0.72976 (3)	0.0597 (4)	

### Atomic displacement parameters (Å^2^)

**Table:**

	*U*^11^	*U*^22^	*U*^33^	*U*^12^	*U*^13^	*U*^23^
C1	0.061 (6)	0.043 (4)	0.037 (4)	0.004 (5)	−0.002 (5)	−0.002 (4)
C2	0.060 (6)	0.036 (4)	0.040 (4)	−0.001 (4)	−0.002 (4)	−0.006 (4)
C3	0.040 (4)	0.039 (4)	0.034 (4)	−0.008 (4)	−0.002 (3)	0.004 (3)
C4	0.034 (4)	0.038 (4)	0.038 (4)	0.000 (3)	−0.004 (3)	−0.001 (3)
C5	0.039 (4)	0.036 (4)	0.029 (4)	0.007 (3)	0.003 (3)	−0.002 (3)
C6	0.045 (5)	0.040 (4)	0.034 (4)	−0.003 (4)	−0.009 (4)	0.001 (3)
C7	0.030 (4)	0.039 (4)	0.039 (4)	−0.002 (4)	−0.002 (4)	0.006 (3)
C8	0.028 (4)	0.034 (4)	0.036 (4)	−0.004 (3)	0.000 (3)	−0.003 (3)
C9	0.033 (4)	0.037 (4)	0.032 (4)	0.007 (3)	0.004 (3)	0.005 (3)
C10	0.056 (6)	0.048 (4)	0.044 (4)	−0.013 (4)	−0.004 (4)	−0.001 (4)
C11	0.072 (6)	0.040 (4)	0.058 (5)	−0.001 (5)	−0.024 (5)	−0.011 (4)
N1	0.049 (5)	0.049 (4)	0.045 (5)	0.001 (4)	0.001 (4)	−0.001 (4)
N2	0.037 (4)	0.046 (4)	0.058 (5)	0.001 (3)	−0.012 (4)	0.001 (4)
O1	0.031 (3)	0.049 (3)	0.042 (3)	0.010 (2)	−0.003 (2)	−0.002 (3)
O2	0.071 (4)	0.063 (4)	0.061 (4)	0.026 (4)	0.003 (3)	−0.013 (3)
O3	0.074 (5)	0.092 (5)	0.088 (5)	0.039 (4)	−0.010 (4)	−0.023 (4)
O4	0.102 (6)	0.076 (4)	0.059 (5)	0.023 (4)	0.012 (4)	−0.014 (4)
O5	0.060 (5)	0.130 (7)	0.108 (6)	0.004 (4)	−0.033 (5)	0.048 (5)
O6	0.038 (4)	0.160 (8)	0.141 (8)	−0.011 (5)	−0.002 (5)	0.073 (7)
Br	0.0621 (6)	0.0560 (5)	0.0611 (6)	0.0056 (5)	−0.0181 (5)	−0.0042 (4)

### Geometric parameters (Å, °)

**Table:**

C1—O2	1.194 (9)	C7—C9	1.397 (10)
C1—O1	1.398 (9)	C7—N2	1.471 (10)
C1—C2	1.438 (11)	C8—C9	1.382 (9)
C2—C3	1.342 (10)	C9—O1	1.368 (8)
C2—H3	0.9300	C10—H11A	0.9700
C3—C8	1.458 (10)	C10—H11B	0.9700
C3—C10	1.494 (11)	C11—H12A	0.9600
C4—C8	1.370 (10)	C11—H12B	0.9600
C4—C5	1.383 (9)	C11—H12C	0.9600
C4—H5	0.9300	N1—O3	1.169 (9)
C5—C6	1.398 (10)	N1—O4	1.210 (9)
C5—N1	1.488 (10)	N2—O5	1.160 (8)
C6—C7	1.375 (10)	N2—O6	1.201 (9)
C6—C11	1.501 (10)		
			
O2—C1—O1	116.2 (8)	C9—C8—C3	117.3 (7)
O2—C1—C2	127.4 (8)	O1—C9—C8	122.8 (7)
O1—C1—C2	116.3 (7)	O1—C9—C7	116.4 (6)
C3—C2—C1	123.1 (7)	C8—C9—C7	120.9 (7)
C3—C2—H3	118.4	C3—C10—Br	108.9 (5)
C1—C2—H3	118.4	C3—C10—H11A	109.9
C2—C3—C8	119.2 (7)	Br—C10—H11A	109.9
C2—C3—C10	120.9 (7)	C3—C10—H11B	109.9
C8—C3—C10	120.0 (7)	Br—C10—H11B	109.9
C8—C4—C5	121.6 (7)	H11A—C10—H11B	108.3
C8—C4—H5	119.2	C6—C11—H12A	109.5
C5—C4—H5	119.2	C6—C11—H12B	109.5
C4—C5—C6	122.9 (7)	H12A—C11—H12B	109.5
C4—C5—N1	116.5 (7)	C6—C11—H12C	109.5
C6—C5—N1	120.7 (7)	H12A—C11—H12C	109.5
C7—C6—C5	114.4 (7)	H12B—C11—H12C	109.5
C7—C6—C11	120.8 (7)	O3—N1—O4	125.5 (8)
C5—C6—C11	124.8 (7)	O3—N1—C5	118.9 (7)
C6—C7—C9	123.3 (7)	O4—N1—C5	115.6 (7)
C6—C7—N2	120.2 (7)	O5—N2—O6	123.2 (8)
C9—C7—N2	116.5 (6)	O5—N2—C7	119.7 (8)
C4—C8—C9	116.9 (7)	O6—N2—C7	117.0 (7)
C4—C8—C3	125.8 (7)	C9—O1—C1	121.2 (6)
			
O2—C1—C2—C3	179.1 (8)	C3—C8—C9—O1	1.3 (10)
O1—C1—C2—C3	−2.9 (11)	C4—C8—C9—C7	0.4 (11)
C1—C2—C3—C8	2.1 (11)	C3—C8—C9—C7	−179.7 (6)
C1—C2—C3—C10	−176.6 (7)	C6—C7—C9—O1	177.6 (6)
C8—C4—C5—C6	−3.6 (11)	N2—C7—C9—O1	−4.9 (9)
C8—C4—C5—N1	177.2 (7)	C6—C7—C9—C8	−1.5 (11)
C4—C5—C6—C7	2.5 (11)	N2—C7—C9—C8	176.0 (7)
N1—C5—C6—C7	−178.4 (6)	C2—C3—C10—Br	104.6 (7)
C4—C5—C6—C11	−175.8 (7)	C8—C3—C10—Br	−74.1 (7)
N1—C5—C6—C11	3.3 (11)	C4—C5—N1—O3	41.9 (11)
C5—C6—C7—C9	0.0 (10)	C6—C5—N1—O3	−137.3 (8)
C11—C6—C7—C9	178.4 (7)	C4—C5—N1—O4	−136.2 (8)
C5—C6—C7—N2	−177.3 (6)	C6—C5—N1—O4	44.6 (10)
C11—C6—C7—N2	1.0 (11)	C6—C7—N2—O5	−81.1 (10)
C5—C4—C8—C9	2.0 (11)	C9—C7—N2—O5	101.4 (9)
C5—C4—C8—C3	−177.9 (7)	C6—C7—N2—O6	101.1 (10)
C2—C3—C8—C4	178.7 (7)	C9—C7—N2—O6	−76.4 (10)
C10—C3—C8—C4	−2.6 (11)	C8—C9—O1—C1	−2.2 (10)
C2—C3—C8—C9	−1.2 (10)	C7—C9—O1—C1	178.7 (6)
C10—C3—C8—C9	177.5 (7)	O2—C1—O1—C9	−178.9 (7)
C4—C8—C9—O1	−178.6 (6)	C2—C1—O1—C9	2.9 (10)
